# Stress Classification Using Photoplethysmogram-Based Spatial and Frequency Domain Images

**DOI:** 10.3390/s20185312

**Published:** 2020-09-17

**Authors:** Sami Elzeiny, Marwa Qaraqe

**Affiliations:** Information & Computing Technology, College of Science & Engineering, Hamad Bin Khalifa University, P.O. Box: 34110, Education City, Doha, Qatar; mqaraqe@hbku.edu.qa

**Keywords:** stress status, convolution neural network, PPG signal, spatial domain, frequency domain, image processing

## Abstract

Stress is subjective and is manifested differently from one person to another. Thus, the performance of generic classification models that classify stress status is crude. Building a person-specific model leads to a reliable classification, but it requires the collection of new data to train a new model for every individual and needs periodic upgrades because stress is dynamic. In this paper, a new binary classification (called stressed and non-stressed) approach is proposed for a subject’s stress state in which the inter-beat intervals extracted from a photoplethysomogram (PPG) were transferred to spatial images and then to frequency domain images according to the number of consecutive. Then, the convolution neural network (CNN) was used to train and validate the classification accuracy of the person’s stress state. Three types of classification models were built: person-specific models, generic classification models, and calibrated-generic classification models. The average classification accuracies achieved by person-specific models using spatial images and frequency domain images were 99.9%, 100%, and 99.8%, and 99.68%, 98.97%, and 96.4% for the training, validation, and test, respectively. By combining 20% of the samples collected from test subjects into the training data, the calibrated generic models’ accuracy was improved and outperformed the generic performance across both the spatial and frequency domain images. The average classification accuracy of 99.6%, 99.9%, and 88.1%, and 99.2%, 97.4%, and 87.6% were obtained for the training set, validation set, and test set, respectively, using the calibrated generic classification-based method for the series of inter-beat interval (IBI) spatial and frequency domain images. The main contribution of this study is the use of the frequency domain images that are generated from the spatial domain images of the IBI extracted from the PPG signal to classify the stress state of the individual by building person-specific models and calibrated generic models.

## 1. Introduction

Stress is a mental, emotional, and physical reaction experienced when a person perceives demands that exceed their ability to cope. The two common forms of stress are acute stress and chronic stress. Acute stress is a short-term form and caused by recent past and near future demands, events, or pressures. Money worries, losing a job, causing an accident, taking an exam, death of a close family member, serious injury, or attending an interview can cause acute stress disorder. However, it requires a relief technique to relax and recover, such as breathing exercises, get outdoors, or muscle relaxation. In contrast, chronic stress is a long term form and resulting from prolonged and repeated exposure to stressors for a prolonged period and can lead to more severe health problems if it is not handled adequately [1,2,3]. Chronic stress weakens the body’s immune system, leading to several mental and physical illnesses such as depression and cardiovascular diseases [4]. People experience acute stress several times throughout the day, and as the symptoms in acute stress are more evident than in chronic stress, the acute stress has been widely researched. Approximately 265 million people worldwide suffer from depression and lost productivity caused by the anxiety, and depression costs the global economy US$ 1 trillion per year [5]. According to a survey, more than 75% of American adults report emotional or physical signs of stress. Moreover, during the COVID-19 pandemic, stress levels increased for half of parents with children under the age of 18 due to stressors related to education and basic needs [6]. Consequently, it is essential to detect stress in its early phase to avoid more damages, prevent it from being chronic, and provide stressed people with stress management strategies. Several techniques are used to measure stress either in real-life or theoretically. Psychological questionnaires usually suffer from response and memory biases when the memory recall period is long. Invasive methods exist, such as measuring cortisol levels in saliva, blood, urine, and hair [7,8]. Noninvasive methods measure physical and physiological responses, such as using facial expressions, eye gaze, electrocardiogram (ECG), photoplethysmogram (PPG), galvanic skin response (GSR), and electroencephalography (EEG). Invasive methods require the use of research laboratories with special and expensive types of equipment. On the other hand, noninvasive techniques are performed using relatively simple devices. Thus, they are easy and more popular among researchers. During stress, the sympathetic branch of the autonomic nervous system (ANS) is hyperactivated, which directs the body’s response, e.g., by sending information to the cardiovascular system to boost heart rate, which can be measured by ECG or PPG signals. ECG sensors use electrical signals generated by heart activity, while PPG sensors use a photodetector and light source to measure blood volume changes per pulse. The variation in time between consecutive heartbeats is called heart rate variability (HRV) and is used as a marker for ANS activity and a psychological stress indicator. A stressed heart beats faster than usual and the HRV decreases. Pulse rate variability (PRV) extracted from the PPG signal high correlates with HRV and can be used as an alternative stress measure [9,10,11,12,13,14,15,16]. ECG devices are non-portable and often unavailable. Thus, smartphones and wrist-worn PPG embedded sensors are used as alternatives to ECG to monitor stress. Several devices, including medical, smartphone, and wearable, have been developed to detect and monitor stress through electrode–skin contact methods or smartphone camera-based PPG [16,17,18,19,20].

Machine learning (ML) and deep learning (DL) techniques are used in stress recognition. ML can achieve a good classification or prediction accuracy in the detection of stress status. However, it requires domain knowledge and expertise to engineer, and it is necessary to extract the optimal features to reduce the complexity of the raw data and make patterns easier for the algorithm to learn. In contrast, DL methods send raw data through different layers in which specific features are defined, and more complex nonlinear patterns and hierarchical information are learned. Wonju et al. proposed a modified network structure based on Deep ECG Net. Wonju et al. utilized ECG and respiration (RESP) signals and achieved 83.9% average classification accuracy to detect workplace stress [21]. Youngjun et al. proposed a deep learning model called deep breath. It recognizes the stress level through breathing patterns and achieved 84.5% accuracy in distinguishing between two stress levels [22]. The authors of [23] applied a deep neural network (DNN) and transfer learning method on raw ECG signals for binary classification between stress and rest state of the driver. Sabyasachi et al. developed a multichannel deep learning architecture to detect stress by using ECG, reparation, and electromyography (EMG) data from the chest using the RspiBan device and triaxial accelerometer, BVP and temperature signals from the wrist using Empatica E4 device. Their network promoted an average precision and recall of 97.6% and 97.2%, respectively [24]. In computer vision and ML, the Fourier domain is used to increase the speed of the training time without losing efficiency, especially for large images, which take a long time and need more memory consumption using a convolution neural network (CNN). Bochen et al. developed a spectral domain CNN (SpecNet) which uses convolution and spectral-domain activation operations to reduce memory consumption by around 60% without reducing the performance of all tested CNN architectures (VGG, LeNet, DenseNet, and AlexNet) [25]. Harry et al. proposed a Fourier convolution neural network (FCNN), in which training is conducted in the Fourier domain. The results indicated that convolution in the Fourier domain speeds up without affecting the accuracy of image classification [26]. Faster and more accurate image classification was obtained by Fourier-based convolution neural network (FCNN). Quan Liu et al. designed CNN models to predict the depth of anesthesia (DOA) indicator for patients from the EEG-based spectrum, and the model achieved 93.5% and can provide physicians with measures to prevent the influence of patient and anesthetic drug differences [27]. Koln et al. developed several neural networks to classify images in the Fourier domain to visualize patterns learned by the networks, and they found the important regions to classify particular objects [28]. Frequency domain features are important for image classification as well as spatial features, especially when the spatial resolution increases [29]. Lin et al. classified pixels in frequency domain infrared microscopic images to human breast cell and non-cell categories by K-means clustering [30]. However, perceived stress is very subjective and expressed differently among different people. The generic model can classify stress status for the unseen person, but the stress classification model needs personalization due to the differences in individual stress responses to stress and coping ability. Moreover, a stressful situation for one individual may not be an issue for another one, and females will, in general, have a higher level of stress than men. Likewise, there exist differences in stress vulnerability, reactivity, resilience, and responsiveness to the threading events. Therefore, building a person-specific classification model is significant [31,32,33]. Martin et al. found that developing student-specific models yielded better results than general and cluster-specific classification models for perceived stress detection in students using smartphone data [34]. Kizito et al. proposed a hybrid stress prediction method, which revealed an increase in generic model accuracy from 42.5% to 95.2% by combining 100 person-specific samples used to train the generic model. They tested their new approach on two different datasets and found that the calibrated stress detection model outperformed the generic one. Jing et. al proposed a new classification model for the drive’s stress level using IBI images for the ECG signal and CNN. They compared the accuracy of this approach with the ANN method using time-domain features (mean IBI and root mean squared difference of adjacent IBIs (RMSSD), and standard deviation of IBIs (SDNN)). They found that the accuracy of the new approach was more accurate than the ANN method, which has been frequently used in recent researches [35].

In this study, a new stress classification approach is proposed to classify the individual stress state into stressed or non-stressed by converting spatial images of inter-beat intervals of a PPG signal to frequency domain images and we use these pictures to train several CNN models. Three types of stress classification models were used: person-specific models, generic models, and calibrated-generic models taking into account intra-individual and interindividual differences. The accuracy measurements of the proposed models (person-specific, calibrated generic model) showed the potential of using frequency domain images in stress detection. Our binary classification approach can be applied to classify the state of the daily life stress of individuals into stressed or non-stressed using inter-beat intervals (IBIs) data. Moreover, it can be used to to monitor a person’s psychological wellbeing in everyday life and trigger clinical intervention when the occurrence of acute stress states detected in a specific patient becomes too frequent. This could prompt the clinician to look for lifestyle related issues at the origin of the stress.

This paper is structured as follows. Section 2 describes the dataset used in this research, and Section 3 describes the proposed stress image-based detection model. In Section 4, the results for the proposed models are discussed. Section 5 presents the results and states the findings of this research.

## 2. Materials and Data

Wearable stress and affect detection (WESAD) is a publicly available data set that contains motion and physiological data recorded from the chest and wrist-worn devices and self-reports of 15 subjects in laboratory settings during three conditions (baseline, amusement, and stress) [36]. The WESAD multimodal data was used for this study. The Tier Social Stress Test (TSST) was implemented for inducing psychological stress [37]. The TSST is a procedure that induces acute social stress in a laboratory environment. In TSST, the public speaking is followed directly by mental math task in the same session, both are delivered in front of an interview panel, and both introduce novelty and uncontrollably [38]. In the baseline session, the subjects were given a neutral magazine to read for 20 min and watched a set of funny movies for amusement. While in stress conditions, they were exposed to public speaking and mental arithmetic tasks. The participants delivered a five-minute speech in front of a panel and were then asked to count down numbers from 2023 to zero with 17 steps. A repeat count was mandated for any mistake in the course of the counting exercise. For mediation, the subject performed a controlled berating exercise, during which PPG, ECG, EMG, EDA, skin temperatures, acceleration, and respiration signals were recorded using RespiBAN Professional and Empatica E4. RespiBan recorded ECG, EMG, EDA, Temp, ACC, and RESP data sampled at 700 Hz. E4 records EDA (4 Hz), ACC (32 Hz), BVP (64 Hz), and Temp (4 Hz). The data collection was conducted in a laboratory setting. In this study, the IBI sequence provided by Empatica E4 wristband was used. IBI is computed by using a proprietary algorithm provided by Empatica to detect heartbeats from the BVP signal and calculated the lengths of the intervals between adjacent beats. In Empatica E4, the BVP signal is collected by a PPG sensor using a proprietary algorithm that combines the light signals detected during the exposure of the red and green lights with a 64 Hz sampling rate. The IBI data file consists of two columns: a timestamp and the duration of the detected beats. The incorrect peaks caused by noise in the BVP signal were removed from the file [39,40]. The IBI data for the public speaking and mental math task were combined to reflect the stress class to build a binary classification model to classify the stress state of a person into two categories: stressed or non-stressed.

## 3. Proposed Stress Image-Based Detection Model

**Figure 1:**
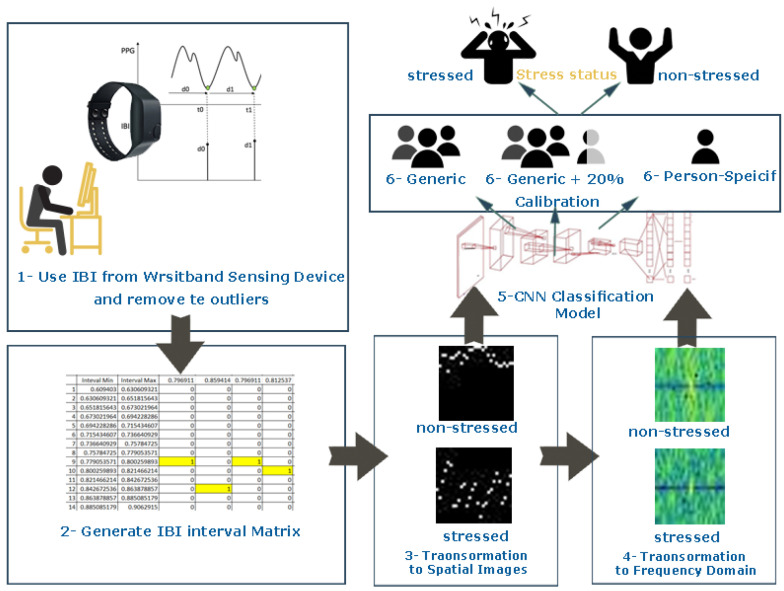
Proposed stress classification models development.

The IBI is a significant cardiac measure, which is used to detect stress and provides an emotional state of the individual [17,41]. In this paper, the entire time interval of the extracted IBI data from PPG signals was divided into intervals according to their distributions. n×m matrices determine inter-beat interval distribution. After that, spatial images were generated from the extracted matrices and converted to frequency domain images for stress classification models using deep convolutional neural networks. The output of the classification model is the stress state of the person (stressed or non-stressed) as shown in Figure 1.

### 3.1. Spatial Domain Image Generation

**Figure 2:**
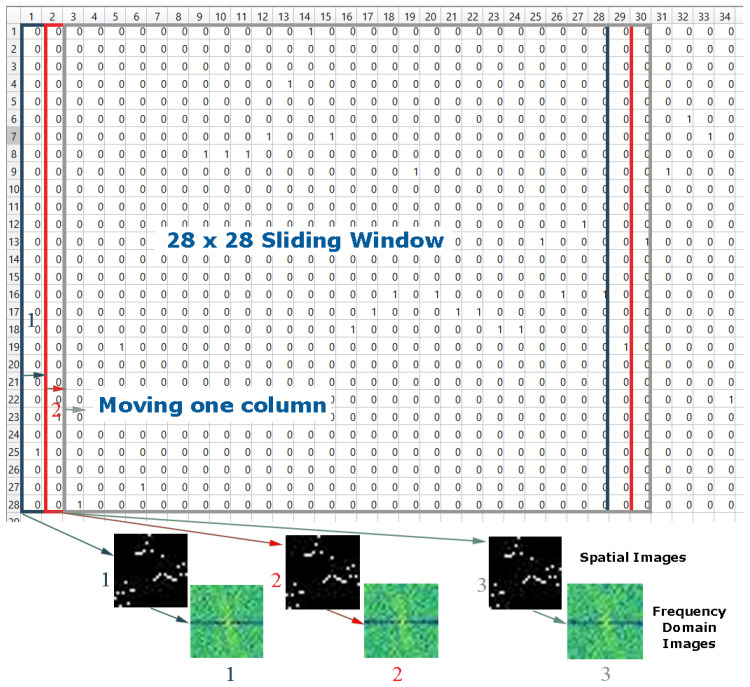
Sliding window of size 28 with overlapping by one column.

**Figure 3:**
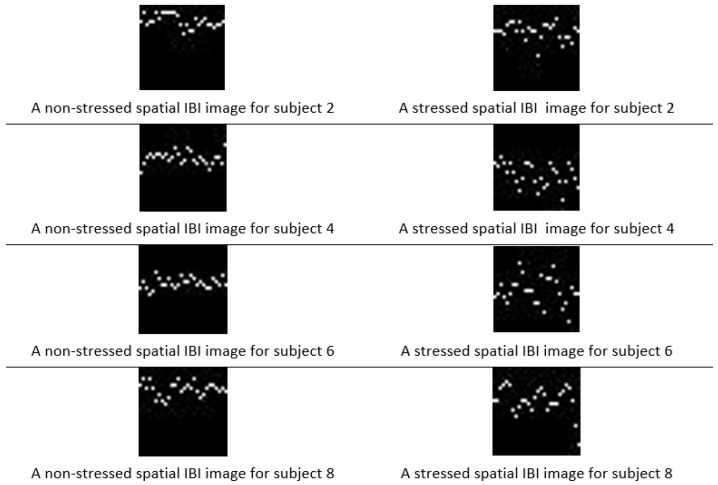
Stressed and non-stressed spatial images for several subjects.

An image can be presented as a 2D matrix, and each element in this matrix represents pixel intensity. The intensity distribution of the image is called a spatial domain. For the colored image, the spatial domain can be described as a 3D vector of 2D matrices that contains the intensities for RGB colors. The abnormal values outside the IBI normal ranges (6–1.2 s) were removed. Then, the descriptive statics were calculated, such as range, minimum, and maximum values, and the time interval of the inter-beat interval was divided into 28 intervals according to the distribution of the inter-beat intervals as discussed in [35]. Second, a N×1 column vector was created for each inter-beat interval and assigned 1 to the interval in which the inter-beat belongs and 0 for the remaining elements. Third, an n×m matrix was formed by concatenating the consecutive m column vectors, transferring the output matrix to 28 × 28 pixel spatial domain images using MATLAB. A sliding window of size 28 was moved only with the column, as shown in Figure 2. Figure 3 shows two different images for several subjects in both condition stressed and non-stressed state.

The value of pixel intensity is the primary information stored in the pixels, and the most significant feature used for image classification. The intensity of an image is the mean of all pixels in the image. The average pixel intensity was calculated for non-stressed and stressed images in order to quantify the differences between the two classes using the generated images. Table 1 shows the mean of all the pixel values in the entire image for several subjects in both conditions (stressed and non-stressed) are shown. Table 2 displays the average intensity for the four segments of each image in two different conditions (stressed and non-stressed). The mean values of the stress images are higher than the non-stressed spatial images.

### 3.2. Frequency Domain Image Generation

A spatial image can be represented in a frequency domain using transformation. In the output image, each point represents a particular frequency contained in the spatial domain image. In the frequency domain image, high- and low-frequency components correspond to edges and smooth regions, respectively; such image transformation helps to reveal pixels information and detect whether or not repeating patterns exist. The Fourier transform is utilized to decompose a spatial domain equivalent image into its cosine and sine components. For a squared image of size n×m pixels, the 2D Discrete Fourier Transform (DFT) is given by the Equation (1), in which the value of each point F(u,v) is calculated by the summation result of multiplying the spatial image with the corresponding base function.

(1) $$F\left(u,v\right)=\frac{1}{MN}\sum_{x=0}^{M-1}\sum_{y=0}^{N-1}f\left(x,y\right)e^{-j2\pi (ux/M+vy/N)}$$

where

*u* = frequency in x-direction, *u* = 0, …, *M* − 1
*v* = frequency in y-direction, *v* = 0, …, *N* − 1

**Figure 4:**
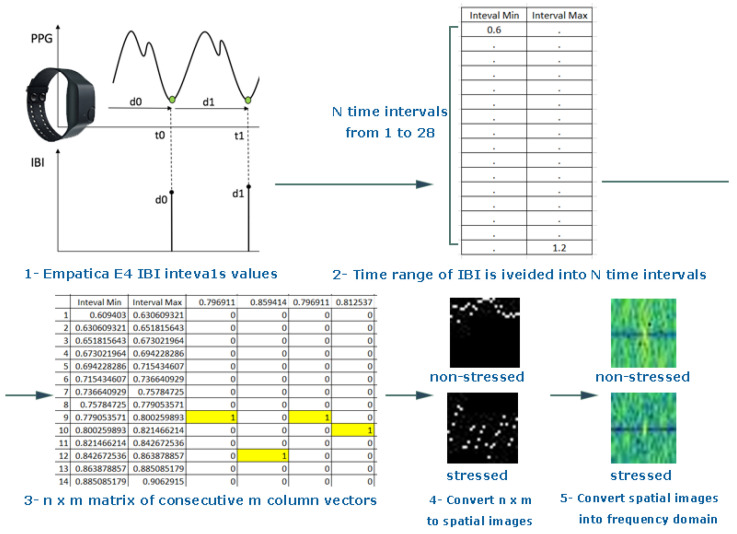
Converting inter-beat intervals (IBI) obtained from the photoplethysmogram (PPG) signal into frequency domain images.

In this research, the spatial image is converted to the frequency domain by applying fast Fourier transformation (FFT) on spatial images to get the frequency domain version for these images based on Algorithm 1 as shown in the Figure 4. Classification performance in the Fourier domain outperforms the classification in the spatial domain [28,42,43]. Moreover, image processing using frequency domain images provides more features and reduces the computational time of the classification model. In addition, image in frequency domain offers another level of information that spatial domain images can not provide. Specifically, frequency domain images provide information with the rate at which the pixel values are changing in spatial domain. The rate (frequency) of this change has information that can be exploited to enhance classification models. The FFT is a fast algorithm that is used to compute the DFT. DFT computation takes approximately $N^{2}$ (DFT computational complexity: $O(N^{2}$)) whereas FFT computation takes approximately $N\log\left(N\right)$ (FFT computational complexity: $O\left(N\log\left(N\right)\right)$)
**Algorithm 1:** Transforming IBI spatial images into frequency domain images
 **Input:** IBI spatial image 
 **Data:** Image data $Img_{data}$ as array
 **Output:** IBI frequency domain image 
 /* apply 2-D fast Fourier transform 
f← np.fft.fft2($Img_{data}$); 
f← np.fft.fftshift(f); 
f← np.abs(f); 
f← np.$\log_{10}\left(f\right)$; 
/* find the original contrast range 
high← np.nanmax(fourier[np.isfinite(f)]); 
min← np.nanmin(fourier[np.isfinite(f)]); 
orgContImg← high-min; 
/* transofrm normalized data into an image 
normFourier ← (f-min) / orgContImg * 255; 
normFourierImg ← Image.fromarray(normFourier); 
**return**
*normFourierImg*; 
/* Save image as a file 
matplotlib.image.imsave(flname, normFourierImg,cmap = viridis)*/ */ */ */

**Figure 5:**
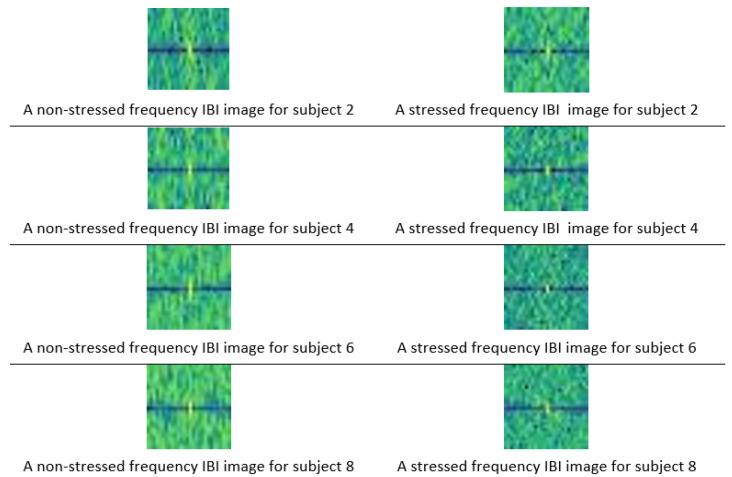
Stressed and non-stressed frequency domain images for several subjects.

Figure 5 displays IBI images in the frequency domain for different subjects in stressed and non-stressed conditions.

Table 3 shows the average pixel intensity for the frequency domain images for subjects in the two conditions: stressed and non-stressed. The mean values of the stressed IBI frequency domain images are lower than the non-stressed images.

### 3.3. Deep Learning-Based Classification

**Figure 6:**
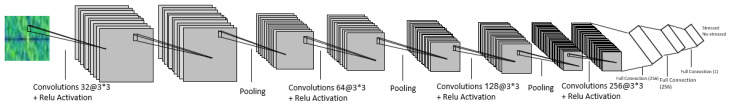
Stress classification using a convolution neural network (CNN) model structure.

CNN is an example of a deep learning neural network and can be used for computer vision tasks such as image classifying by processing the input image and output the class or probability that the image belongs to it. CNN has input, output, and hidden layers in which it extracts features from images while the network trains on a set of pictures. It applies several filters on the input image to build the feature map and trains through forward and back-propagation for many epochs until reaching a distinct network with trained weights and features. To classify individual stress status into stressed or non-stressed, a 19-layer CNN model was built, as illustrated in Figure 6. CNN is a deep learning algorithm used for image classification and object detection. Images pass through 2D convolution layers with kernels, pooling, and fully connected layers. CNN extracts features from the input images while the network trains, and each layer increases the complexity of learned features. Like other artificial neural networks, CNN or ConvNet has an input, several hidden layers (e.g., convolution layers), and an output layer. Convolution is a linear operation that includes the multiplication of a set of 2D weight arrays called the filter or kernel with the input data array. The output of this multiplication is a 2D array called feature map. The feature map values are passed through nonlinear functions, such as the rectified linear unit (ReLU). CNN can train and learn abstract features for efficient object identification. It does not suffer from overfitting, overcomes the limitation of other machine learning algorithms, and is very effective at reducing the parameters amount using dimensional reduction methods without affecting the quality of models. It is used to solve complex problems in different domains such as image classification and object detection due to their better performance [44,45,46,47,48]. In our model, the input image with size of 28 × 28 pixels goes through 8 convolution layers to produce 32, 64, 128, 256 feature maps using filters with a convolution kernel of a 3 × 3 receptive field. There are 4 max-pooling layers with size 2 × 2 after every two convolution layers. Max-pooling is used to reduce the two dropout layers with a rate of 0.5 for regularization. The fully connected layers have depths of 256, 256 and 1. ReLU activation layers are used to increase nonlinearity in the network. The outputs of these networks were stressed and non-stressed.

The following stress classification models were trained, tested, and evaluated using our CNN model architecture and both type of images (spatial and frequency domain).

1. Person-specific models using spatial images: Models were trained, validated, and tested on the spatial domain images of the same subject. The entire datasets were divided into 70%, 15%, and 15% for training, validation, and testing, respectively
2. Person-specific models using frequency domain images: Models were trained, validated, and tested on the frequency domain images of the same subject. The entire datasets were divided into 70%, 15%, and 15% for training, validation, and testing, respectively.
3. Generic models using spatial domain images: Models were trained and validated models on the spatial domain images of 12 subjects (n−3) and we tested their performance on three others that were left out. Of these, three were used to evaluate the model’s accuracy in classifying the unseen person’s stress status. Three subjects were in the test dataset, and the other 12 subjects’ data were in the training and validation sets.
4. Generic models using frequency domain images: The models were trained and validated models on the frequency domain images of n-3 subjects, and we tested their performance on the left out three subjects frequency domain images. Three subjects were in the test dataset, and the other 12 subject’s data were in the training and validation sets.
5. Generic models using spatial domain images with calibration samples: 20% of the test dataset were incorporated in the training pool, and the models were tested on the remaining samples. This approach was implemented because the performance of the generic model is lower than the person-specific model. For models training and accuracy measurements, three subjects of data were used as a test dataset and we combined 20% of these data into the other 12 subjects’ data in the training datasets.
6. Generic models using frequency domain images with calibration samples: 20% of the test dataset was incorporated in the training pool, and the models were tested on the remaining samples. Three subjects’ data were used as a test dataset, and 20% of their data were combined with the training dataset to train the model and measure its accuracy.

The above classification models were evaluated by measuring the accuracy of the training, validation, and testing. Moreover, other parameters were also measured. These are the sensitivity (number of samples were classified by the model as positive among all actual positives), specificity (number of samples were classified by the model as Negative among all actual Negatives), and precision (how many samples were positive among all classified positive samples).

## 4. Results

The inputs were spatial images and frequency domain images, and the output was stressed or non-stressed. The performance of the classification models was measured by comparing the values of accuracy for the train, valid and test, along with the test sensitivity (true positive rate), precision, and specificity (true negative rate). The equations for calculating these performance metrics are shown in Equations (2)–(5). The accuracy is the ratio of the correct classifications from all classifications.

(2) $$Accuracy=\frac{TP+TN}{TP+TN+FP+FN}$$

Sensitivity is defined as the capability of a test to correctly classify a person as stressed:

(3) $$Sensitivity=\frac{TP}{FN+TP}$$

The specificity is the capability of a test to correctly classify a person as non-stressed:

(4) $$Specificity=\frac{TN}{TN+FP}$$

Precision measures how correctly the classifier was able to classify positive out of all positives:

(5) $$Precision=\frac{TP}{TP+FP}$$

The classification accuracy measurements for all models were satisfactory among the training, validation, and test datasets. The person-specific models achieved high performance compared to the generic models. The average classification accuracy of the person-specific models using spatial images for the training, validation, and test datasets was 99.9%, 100%, and 99.8%, respectively. For the person-specific models using frequency domain images, the accuracy was 99.68%, 98.97%, and 96.4%. The performance of the generic models varied between the different subjects and had lower accuracy than the person-specific models. The average accuracy for the generic classification models using spatial images was 98.6% (train), 96.8% (valid), and 61% (test), and 98.9% (train), 97.6% (valid), and 62.6% (test) when using frequency domain images. Moreover, the accuracy for frequency domain classification models was slightly lower than the spatial image classification models, as shown in Table 4 and Table 5. The generic models cannot perfectly recognize the inter-subject difference in response to stress events. Thus, adding some samples from the test to training data significantly increased the accuracy of the generic models as shown in Table 6 and Table 7 when using spatial images and Table 8 and Table 9 when using frequency domain images. By adding these samples, the performance of the models significantly increased from 61% to 88.1% and from 62.6% to 87.6% as happened in the generic models for the test dataset when using the spatial and frequency domain images, respectively. Confusion matrix is a performance measurement that visualizes the performance of the classification model on test data in which the true values are known.

**Figure 7:**
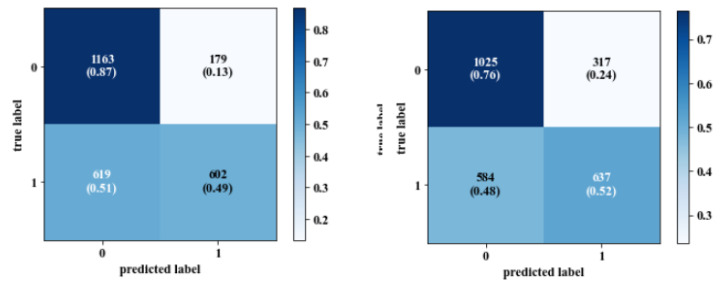
Confusion matrix for generic model (**left**: spatial images; **right**: frequency domain images).

**Figure 8:**
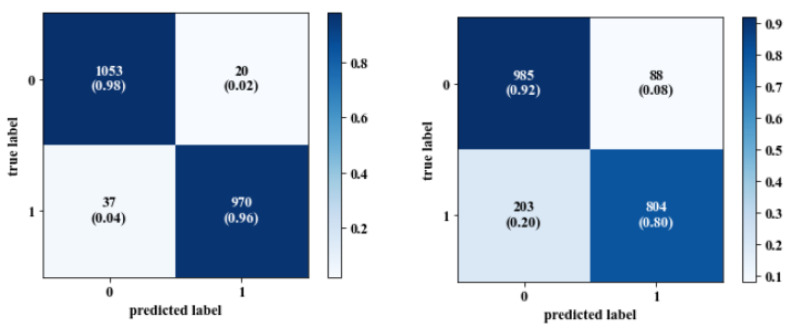
Confusion matrix for generic model using 20% of calibrated sample (**left**: spatial images; **right**: frequency domain images).

The generic model had 179 non-stressed spatial images incorrectly classified as stressed while it had 619 stressed images incorrectly classified as non-stressed, as shown in Figure 7 (left). However, the majority of the spatial images were classified correctly, while by adding 20% of the test data into the training pool, the performance of the model was increased, as it had 20 non-stressed spatial images incorrectly classified as stressed and 37 stressed images incorrectly classified as non-stressed, as shown in Figure 8 (left).

From the confusion matrices in Figure 7 and Figure 8, where the data of subjects 8, 9, and 10 were in the test dataset, and 20% of their calibrated samples were injected in the training dataset, the sensitivity was increased to 96% and 80% and specificity was also increased to 98% and 92% for spatial and frequency domain images, respectively. Adding a few calibration samples allowed the model to learn more information about the unseen person and highlighted the effect of person-specific signals in classifying his/her stress state to either stressed or non-stressed. Another finding is that the time of CNN training and validation using Fourier domain images was lower than that of training and validation on spatial images (e.g., for the person-specific model of he subject number 10, the CNN spent 143 s to train and validate 1019 frequency domain images in around 125 epochs, while using the same number of spatial images took around 214 s). Moreover, to achieve higher accuracy when using spatial and frequency domain images, there is a need to use more epochs to train the generic models. In this study, 150 epochs were used for all generic models using both spatial and frequency domain images.

Table 10 compares the results of this approach with other approaches conducted in the domain of stress detection. One of the main differences between this study and the other studies is the type of images that were utilized for training and validating the models. Moreover, the accuracy of the calibrated model outperformed that of the generic model. Compared to other approaches, the proposed method achieved high accuracy in person-specific models and comparative scores with the other generic models taking into account the different types of the data used (IBI extracted from PPG signal, spatial, and frequency domain images from the IBI) in our study. The results show the potential of using frequency domain images in stress detection.

## 5. Discussion

In this study, a new approach was proposed to classify a person’s stress state using a convolution neural network, spatial, and frequency domain images for inter-beat intervals extracted from the PPG signal. The entire time interval of the extracted IBI data from PPG signals was divided into intervals according to the IBI distributions, and then the output matrix was converted to spatial images. These images were transformed into the frequency domain by using the Fourier transform. Frequency domain features are important for image classification as well as spatial features, especially when the spatial resolution increases. Several types of binary classification models were developed: generic model, person-specific model, and calibrated generic models. The proposed models utilized the IBI’s files generated by Empatica E4 devices founded in the WESAD dataset. The average accuracy for the proposed models achieved a satisfactory performance. The person-specific models were able to classify stress status with high accuracy. Although these models cannot be generalized, it is necessary and effective to personalize the model, as stress is subjective and each person has unique responses and degree of vulnerability to stress. These models can be used in the health monitoring system to monitor the stress status of the patient and can be enriched by collecting new data and training the models again. An image can be represented as a 2d matrix where each element shows pixel intensity. This spatial image can be transformed into the frequency domain by using a Fourier transform. Frequency domain features are important for image classification as well as spatial features, especially when the spatial resolution increases. Images processing using frequency domain images can perform better than spatial domain images, provide more features, and reduce the computation time. CNN is an example of deep learning neural networks and can be used for computer vision tasks such as image classifying by processing the input image and output the class or probability that the image belongs to. CNN has input, output, and hidden layers in which it extracts features from images while the network trains a set of pictures. It applies several filters on the input image to build the feature map and trains through forward and back-propagation for many epochs until it reaches a distinct network with trained weights and features. In this study, a novel approach to classify the stress state of a person by using both spatial and frequency domain IBI images and convolution neural networks is proposed. The proposed models using the IBI’s files generated by Empatica E4 devices founded in the WESAD dataset were tested. Several classification models were built: person-specific, generic, and calibrated generic models. Generic models performed more poorly than the person-specific models when trying to classify stress state of unseen people, as shown in the test accuracy measures in Table 6 and Table 8. These generic models cannot generalize well as stress is subjective, and some people more reactive to stress and have different types of physical and physiological responses. A personalized model was derived by combining a few person-specific samples with the training data to improve the performance of these generic models. In this study, 20% of the subject’s data in the test dataset were combined with the training data, which showed a substantial improvement in the stress classification models performance, as shown by the accuracy measurement in Table 7 and Table 9. These calibrated generic models introduced the subjects’ identities and characteristics to the models. To ensure that our calibrated models were not suffering from overfitting, we validated these models by using 5-fold cross-validation, which leads to unbiased model performance estimation and tests how the different parts of the training set performed in the model. Moreover, the results show that the average accuracy for the generic classification models using frequency domain images was slightly higher than the other models that used spatial images for training, validation, and testing. In addition, classifying the stress status using frequency domain images performed well and provided more features about the entire images and reduced the computation time. The proposed models in this study were effective at classifying stress state and applicable in a stress monitoring health system. Our approach can be applied to monitor a person’s psychological wellbeing and classify his state of daily life stress using inter-beat intervals (IBIs) data. In addition, it can trigger alerts that can be used to guide clinical interventions to prevent and treat symptoms of acute stress disorders when the occurrence of acute stress states detected in a specific patient becomes too frequent. Moreover, stress detection models can be used in the military or police to detect when soldiers and police officers experience high levels of stress that are abnormal and to improve their performance in stressful environments. They can also be used for a student in educational systems to identify which subjects may present issues for particular students. This will enable teachers to intervene and present the material in an alternative manner and minimize stressful events in the classroom as much as they can to reduce stress or anxiety. One limitation of this study is that the proposed models in this study are used to classify the state of an individual into two categories: stressed or non-stressed. The model is aim at instantaneous detection of stress via classification of physiological data. The model does not consider the prediction of stress as it is out of the scope of this work. For future work, newly PPG data will be collected either from lab settings or real-life using wrist-worn devices. These data will be used to train and test the proposed models to measure the accuracy and compare the results.

## Figures and Tables

**Table 1:** The mean of all the pixel values in the entire spatial images (image intensity).

Subject No.	Non-Stressed	Image Intensity	Stress	Image Intensity
2	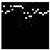	10.52	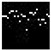	11.40
4	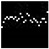	10.36	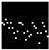	11.04
6	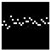	9.94	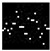	11.54
8	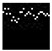	10.81	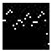	11.43
10	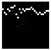	10.27	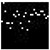	11.31
14	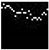	10.60	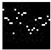	11.28
16	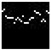	10.69	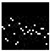	11.24

**Table 2:** Comparing the average intensity value of the image segments in stressed and non-stressed conditions.

Subject	IBI Spatial Image (4 Segments)	Status	Lower- Left, Right	Upper- Left, Right
2	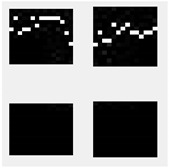	non-stressed	0.2238, 0.2755	19.4933, 19.7000
2	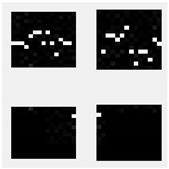	stressed	2.8429, 2.2602	19.2044, 19.5952
16	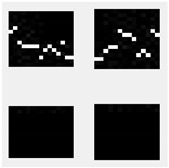	non-stressed	0.4429, 0.4184	19.3200, 20.0667
16	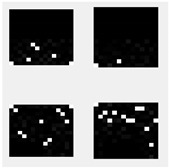	stressed	16.1524, 19.6684	8.7289, 3.9810

**Table 3:** The mean of all the pixel values in the entire frequency images (image intensity).

Subject No.	Non-Stressed	Image Intensity	Stress	Image Intensity
4	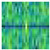	116.90	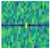	112.89
6	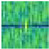	119.91	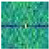	110.95
8	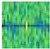	121.71	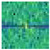	113.71
10	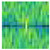	123.10	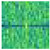	120.81
16	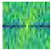	120.26	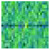	119.73

**Table 4:** The accuracy measures for the person-specific models using spatial images.

Subject No.	Train (%)	Valid (%)	Test (%)	Sensitivity (%)	Specificity (%)	Precision (%)
2	99.8	100	99	98	100	100
3	100	100	99	100	98	98
4	100	100	100	100	100	100
5	100	100	100	100	100	100
6	100	100	100	100	100	100
7	100	100	100	100	100	100
8	100	100	100	100	100	100
9	100	100	100	100	100	100
10	100	100	100	100	100	100
11	100	100	100	100	100	100
13	100	100	100	100	100	100
14	100	100	100	100	100	100
15	100	100	100	100	100	100
16	100	100	100	100	100	100
17	100	100	100	100	100	100
**Average**	**99.9**	**100**	**99.8**	**99.8**	**99.8**	**99.8**

**Table 5:** The accuracy measures for the person-specific models using frequency domain images.

Subject No.	Train (%)	Valid (%)	Test (%)	Sensitivity (%)	Specificity (%)	Precision (%)
2	100	100	97	100	96	96
3	100	100	99	100	98	98
4	100	100	100	100	100	100
5	95.7	84.6	74	100	65	65
6	100	100	93	85	100	100
7	100	100	99	100	99	99
8	100	100	100	100	100	100
9	100	100	99	100	99	99
10	100	100	99	98	100	100
11	99.8	100	99	98	100	100
13	100	100	97	96	100	100
14	100	100	99	100	99	99
15	100	100	96	91	100	100
16	100	100	96	93	97	97
17	99.7	100	99	98	100	100
**Average**	**99.68**	**98.97**	**96.4**	**97.26**	**96.86**	**96.86**

**Table 6:** The accuracy measures for the generic models using spatial domain images.

Subject in Test	Train (%)	Valid (%)	Test (%)	Sensitivity (%)	Specificity (%)	Precision (%)
2,3,4	99.8	99.9	54	43	61	61
5,6,7	99.7	99.8	47	71	34	34
8,9,10	99.7	99.9	69	49	87	87
11,13,14	95.7	84.6	74	100	65	65
15,16,17	99.6	99.8	61	72	52	52
**Average**	**98.6**	**96.8**	**61**	**67**	**60**	**60**

**Table 7:** The accuracy measures for the generic models with 20% calibration samples using spatial domain images.

Subject in Test	Train (%)	Valid (%)	Test (%)	Sensitivity (%)	Specificity (%)	Precision (%)
2,3,4	99.8	99.9	58	63	55	55
5,6,7	99.6	99.9	92	98	88	88
8,9,10	99.7	99.9	97	96	98	98
11,13,14	99.6	100	98	99	96	96
15,16,17	99.6	100	95.5	98	93.5	93.5
**Average**	**99.66**	**99.94**	**88.1**	**90.8**	**86.1**	**86.1**

**Table 8:** The accuracy measures for the generic models using frequency domain images.

Subject in Test	Train (%)	Valid (%)	Test (%)	Sensitivity (%)	Specificity (%)	Precision (%)
2,3,4	98.9	98	56	42	64	64
5,6,7	98.9	97.3	63	61	63	63
8,9,10	98.8	97.6	65	52	76	76
11,13,14	99.1	98	66	45	86	86
15,16,17	98.8	97.4	63	65	60	60
**Average**	**98.9**	**97.66**	**62.6**	**53**	**69.8**	**69.8**

**Table 9:** The accuracy measures for the generic models with 20% calibration samples using frequency domain images.

Subject in Test	Train (%)	Valid (%)	Test (%)	Sensitivity (%)	Specificity (%)	Precision (%)
2,3,4	100	100	97	100	96	96
5,6,7	99	97.6	88	81	92	92
8,9,10	98.9	98.1	86	80	92	92
11,13,14	99.4	97.8	81	96	96	88
15,16,17	98.8	96.2	86	82	89	89
**Average**	**99.22**	**97.94**	**87.6**	**87.8**	**93**	**91.4**

**Table 10:** Comparing the findings of this study with other studies.

Study	Type of Images	Classifier	Type (%)	Accuracy (%)
This Study	PPG-IBI Spatial, Frequency	CNN	Person-specific, Generic	Spatial (99.8,88.1), Frequency (96.4,87.6)
[23]	Raw ECG	CNN	Generic	90.19
[35]	ECG-IBI Spatial	CNN	Generic	92.8
[49]	Face	CNN	Generic	85.23
[22]	Respiration	CNN	Generic	84.59

## Abbreviations

The following abbreviations are used in this manuscript.

IBI	Inter-Beat Interval
PPG	Photoplethysomogram
CNN	Convolution Neural Network
ECG	Electrocardiogram
EEG	Electroencephalography
GSR	Galvanic Skin Response
EDA	Electrodermal Activity
ANS	Autonomic Nervous System
HRV	Heart Rate Variability
DL	Deep Learning
ML	Machine learning
DNN	Deep Neural Network
DOA	Depth Of Anesthesia
FCNN	Fourier Convolution Neural Network
DFT	Discrete Fourier transform
FFT	Fast Fourier transformation
RMSSD	Root Mean Squared Difference of Adjacent IBIs
SDNN	Standard Deviation of IBIs
